# Targeted metabolomic profiling as a tool for diagnostics of patients with non-small-cell lung cancer

**DOI:** 10.1038/s41598-023-38140-7

**Published:** 2023-07-08

**Authors:** Ksenia M. Shestakova, Natalia E. Moskaleva, Andrey A. Boldin, Pavel M. Rezvanov, Alexandr V. Shestopalov, Sergey A. Rumyantsev, Elena Yu. Zlatnik, Inna A. Novikova, Alexander B. Sagakyants, Sofya V. Timofeeva, Yuriy Simonov, Sabina N. Baskhanova, Elena Tobolkina, Serge Rudaz, Svetlana A. Appolonova

**Affiliations:** 1 World-Class Research Center Digital Biodesign and Personalized Healthcare, I.M. Sechenov First Moscow State Medical University, Moscow, Russia 119435; 2 Laboratory of Pharmacokinetics and Metabolomic Analysis, Institute of Translational Medicine and Biotechnology, I.M. Sechenov First Moscow Medical University, Moscow, Russia 119435; 3 I.M. Sechenov First Moscow State Medical University, Moscow, Russia 119435; 4 Pirogov Russian National Research Medical University, Moscow, Russia 117997; 5 National Medical Research Centre for Oncology (Rostov-On-Don, Russia), 14 Liniya, 63, Rostov-on-Don, Russia 344019; 6 Institute of Pharmaceutical Sciences of Western Switzerland, University of Geneva, 1206 Geneva 4, Switzerland

**Keywords:** Cancer metabolism, Lung cancer

## Abstract

Lung cancer is referred to as the second most common cancer worldwide and is mainly associated with complex diagnostics and the absence of personalized therapy. Metabolomics may provide significant insights into the improvement of lung cancer diagnostics through identification of the specific biomarkers or biomarker panels that characterize the pathological state of the patient. We performed targeted metabolomic profiling of plasma samples from individuals with non-small cell lung cancer (NSLC, n = 100) and individuals without any cancer or chronic pathologies (n = 100) to identify the relationship between plasma endogenous metabolites and NSLC by means of modern comprehensive bioinformatics tools, including univariate analysis, multivariate analysis, partial correlation network analysis and machine learning. Through the comparison of metabolomic profiles of patients with NSCLC and noncancer individuals, we identified significant alterations in the concentration levels of metabolites mainly related to tryptophan metabolism, the TCA cycle, the urea cycle and lipid metabolism. Additionally, partial correlation network analysis revealed new ratios of the metabolites that significantly distinguished the considered groups of participants. Using the identified significantly altered metabolites and their ratios, we developed a machine learning classification model with an ROC AUC value equal to 0.96. The developed machine learning lung cancer model may serve as a prototype of the approach for the in-time diagnostics of lung cancer that in the future may be introduced in routine clinical use. Overall, we have demonstrated that the combination of metabolomics and up-to-date bioinformatics can be used as a potential tool for proper diagnostics of patients with NSCLC.

## Introduction

In accordance with the WHO statistics, lung cancer is defined as the second most common oncological disease among both women and men worldwide. In 2020, 2.26 million cases of lung cancer and 1.8 million deaths were recorded worldwide. To date, one of the main reasons for such a high degree of severity of lung cancer is a low level of its diagnosis, associated primarily with the absence of clear clinical symptoms at first stages of the disease, as well as the complexity of pathogenesis. Approximately 80–85% of all cases associated with lung cancer are related to a subtype of non-small cell lung cancer (NSCLC), described in the present study.

It is well known that metabolites play an important role in the occurrence and development of oncological diseases^1–3^. Numerous studies have shown that certain endogenous metabolites or their ratios are effective biomarkers for lung cancer, particularly NSCLC^4,5^. Moreover, abnormalities in the concentration levels of those metabolites may reveal metabolic perturbations in lung cancer patients, reflecting the pathological mechanism of the disease. In this regard, numerous biomedical studies utilize metabolomic tools for the diagnosis of various heterogeneous diseases, including NSCLC^6,7^. Metabolomics is a rapidly emerging field typically used for the identification and quantification of cellular metabolites. Therefore, metabolomic profiling may underline key factors of NSCLC development. Due to the complexity of metabolomic data, optimal information retrieval is usually challenging and therefore up-to-date mathematical and computational approaches^8,9^ for accurate data interpretation are urgently needed.

Traditional statistical methods applied to metabolomics focus on the formation of the relationships among dependent and independent variables, gaining meaningful statistical inferences of the measured variables with regard to the fact that the data are sampled from the larger population^10^. In contrast, machine learning approaches are based on the application of ad hoc computational algorithms that are optimized or learned without the obligatory requirement for formal statistical assumptions^11^. In the present study, we applied supervised ML algorithms to develop the most suitable classification model based on large-scale targeted metabolomic data for NSCLC diagnostics. Additionally, weighted coexpression network analysis^12,13^ together with classical statistical analysis was applied for a deeper clarification of the nonlinear biochemical interconnections between the metabolic changes and appearance of NSCLC, as well as for identification of new significant ratios of the metabolites.

Thus, the main goal of the present study was to perform a deep biochemical interpretation of lung cancer development as well as to create the most suitable ML model for the classification of NSCLC and NC patients based on the results of targeted metabolomic profiling.

## Material and methods

### Study design

The study involved two experimental groups: patients diagnosed with NSCLC at different TNM stages (n = 100) and participants without any malignancies (NC group, n = 100). Informed consent was obtained from all the participants involved in the study. Additional information concerning the characteristics of participants is presented in Table 1.

**Table 1:** Characteristics of the subjects included in the study.

	NSCLC patients	Non-cancer patients
Total number (M/F)	100 (76/24)	100 (21/79)
Age (range)	36–81	23–70
Smoking (%)	45	37
Disease stage (%)	I—6% II—22% III—32% IV—10%	–

The participants selected for the NSCLC group did not undergo antibiotics, pre- and/or probiotic preparations for 3 months and signed an informed consent form. The exclusion criteria for the noncancer participants included age under eighteen, the presence of oncological and severe somatic diseases, diabetes mellitus, any diseases of the gastrointestinal tract, any acute respiratory viral diseases, psychosis, alcoholism, drug addiction, pregnancy and lactation. For the comparative analysis, approximately 2.5 mL of blood sample was drawn in vacutainer tubes before the treatment of all the study participants after overnight fasting. Furthermore, samples were centrifuged at 10,000 rpm to extract serum and stored at -80 °C.

### Ethical approval

The study was approved by ethical committee of Sechenov University (Document #25-20, September 2020) and ethical principles of medical research involving humans stated in the Declaration of Helsinki.

### Chemical reagents

Standard solutions for amino acid, tryptophan metabolism intermediates and acylcarnitine profiling, as well as methanol, formic acid, bovine serum albumin (BSA), sodium chloride, 6-hydroxy nicotinic acid, 3-indole acrylic acid, neopterin, biopterin, 1-tryptophan, and ascorbic acid were received from Sigma‒Aldrich (USA). Acetonitrile was received from Chromasolv® (Sigma‒Aldrich Chemie GmbH, Buchs, Switzerland). Ultrapure water was received through the Millipore Milli-Q purification system (Millipore Corporation, Billerica, MA). Isotope-labeled standard solutions for tryptophan metabolism intermediate profiling were received from Toronto Research Chemicals (USA). Isotope-labeled standard solutions for metabolomic profiling were received from the MassChrom Amino Acids and Acylcarnitines Non Derivatized 57,000 Kit (Chromsystems, Germany).

### Metabolomic profiling

Sample preparation and instrumental analysis for the metabolic profiling of tryptophan metabolites, amino acids, acylcarnitines and NO-cycle metabolite panels were carried out in accordance with the methods presented in the referenced literature^14^. The list of the analyzed metabolites is presented in Supplementary material Table S1.

### Method validation

The applied analytical methods of wide-scale targeted metabolomic profiling were fully validated in accordance with the internal laboratory protocols for validation of bioanalytical methods developed based on the US FDA and EMA guidelines^15,16^. Validation included the assessment of selectivity, linearity, accuracy, precision, matrix effect and stability. Quality control (QC) samples were utilized for the assessment of analysis reproducibility. Calibration parameters were based on the analysis of eight calibrators in three replicates during three analytical runs. Calibration curves were built using weighted linear regression models. Assessment of the inter- and intra- precision and accuracy was performed using QC samples in six replicates during three analytical runs. Stability was assessed using working solutions placed at room temperature (21 ± 3 °C), biological samples placed in an autosampler at (10 ± 0.5 °C) for 24 h and biological samples placed at (35 ± 1 °C) for 20 days. The matrix effect was assessed using QC samples at high and low concentration levels.

### Statistical analysis and development of the machine learning models

Univariate statistical analysis was performed using the Stats package (Python software)^17^. First, the Shapiro–Wilk test was used to check the distribution of the variables. Further analysis of variance was conducted using Student’s T-test or the equivalent nonparametric Mann‒Whitney U-test (p value < 0.05). Additionally, the diagnostic accuracy of the single metabolites was assessed through calculation of the areas under the curve (AUCs) obtained from the receiver operating characteristic curve analyses of the NCSLC and NC groups.

Multivariate analysis, including unsupervised principal component analysis (PCA) and supervised orthogonal partial least squares discriminant analysis (OPLS-DA), was performed using SIMCA software (version 14.0, Umetrics, Umeå, Sweden). The first two principal components (PCs) utilized for the PCA model represented orthogonal transformation of the analyzed metabolites in linearly uncorrelated variables. Based on further OPLS-DA, metabolites with variable importance in the projection (VIP) > 1 were selected as the most discriminative. The combination of the results of the VIP score, P-value and AUC ROC value served for the formation of the preliminarily selected metabolic biomarkers.

Debiased sparse partial correlation (DSPC) network analysis was performed using the desparsified graphical lasso modeling procedure. The modeling strategy is based on the fact that the number of connections between metabolites is less than the available sample size, so the true network of partial correlations is sparse. DSPC rebuilds the graphical model by providing partial correlation coefficients and p values for each pair of features. When using a smaller number of samples, this analysis allows us to detect connections between a large number of metabolites. The results of the analysis are demonstrated in the form of weighted networks. The nodes of this network are metabolites, whereas the edges are partial correlation coefficients or associated P values.

To assess the diagnostic ability of the metabolite combinations, ML-based classification models were developed. To elucidate the most appropriate algorithm and metabolite combination, different ML algorithms were generated using the following:

- absolute concentrations of the preliminarily selected metabolites in NSCLC and NC patient groups
- absolute concentrations of the preliminarily selected metabolites and significant metabolic ratios selected through correlation network analysis in NSCLC and NC patient groups

The datasets were randomly split into training (80%) and validation (20%) datasets using the Stratified K Fold Cross Validation method that served to evaluate the generalization ability of the models. The validation set was utilized for the quality assessment of the models. The assessment was performed using the following quality metrics: accuracy, precision, area under the curve of the receiver operator characteristics (AUCROC), and error matrix. The diagnostic accuracy of the developed ML model was further compared with the top five preliminarily identified biomarkers with the best predictivity values using AUC ROC analysis.

The open-source python scripts are available at https://github.com/FimaLab/lung_cancer.

## Results

**Figure 1:**
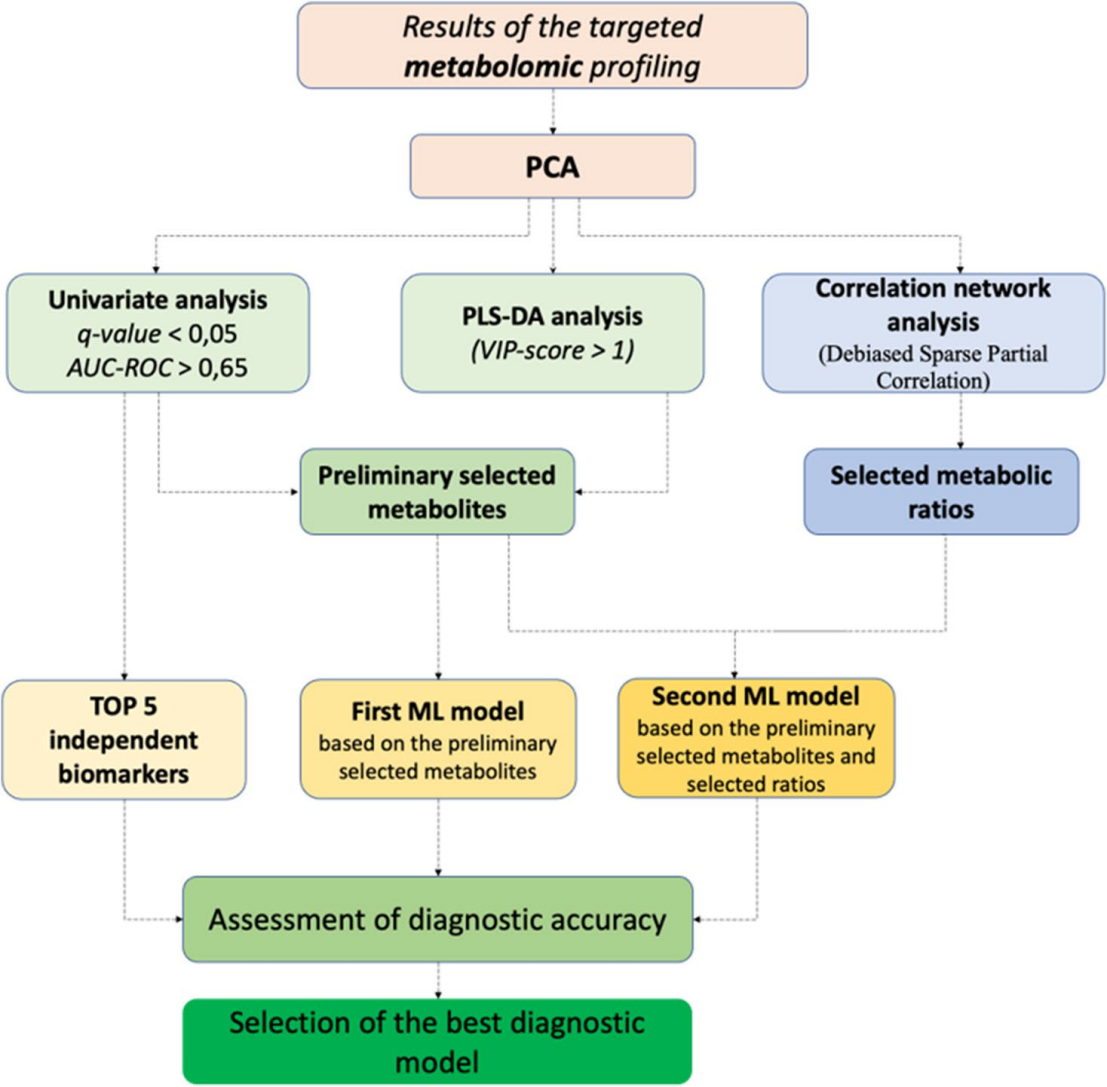
A flowchart depicting the outline of the study, characterizing the performed targeted metabolomic approach and further bioinformatic analysis.

The present study comprised a total of 200 plasma samples from NSCLC and NC patients (Table 1). Figure 1 represents the workflow scheme characterizing the performed targeted metabolomic approach and further bioinformatic analysis that served for the identification of the most significantly altered metabolites as well as for the development of the highly accurate diagnostic model of NSCLC. Metabolic profiling that included the analysis of 63 metabolites was identified through the application of quantitative HPLC‒MS/MS analysis. Furthermore, a global statistical bioinformatic analysis, consisting of correlation network analysis, univariate analysis, multivariate analysis and supervised machine learning modeling used to select the most appropriate classification model, was performed.

### Univariate analysis of the metabolomic results

The final dataset of the received metabolomic profiling results is presented in Supplementary material file 1. Comparison between the NSCLC and NC groups of patients using the nonparametric Mann‒Whitney U-test resulted in the identification of 38 metabolites with p values < 0.05. The box plots of these significantly altered metabolites related to NO cycle intermediates, amino acids, tryptophan metabolism intermediates and acylcarnitine profiles are depicted in Supplementary material, Fig. S1a–d, respectively.

Furthermore, the predictive ability of the individual metabolites in the discrimination of NSCLC and NC patients was assessed using AUC ROC analysis. As a result, twenty-eight metabolites demonstrated AUC scores higher than 0.65 and were considered significant. The highest AUC values were obtained for ADMA, SDMA, aspartic acid, C6-DC, and tryptamine (AUC > 0.75), which were further utilized as individual diagnostic markers.

Based on the results of the full profiling, an unsupervised PCA was constructed. Thus, a four-component PCA model was obtained with the parameters stated as follows: R2X = 0.398; Q2 = 0.145. The PCA score plot (Supplementary material, Fig. S2) shows relative separation between the patient groups. It interprets general differences in the metabolomic profiles between NSCLC and NC groups.

**Figure 2:**
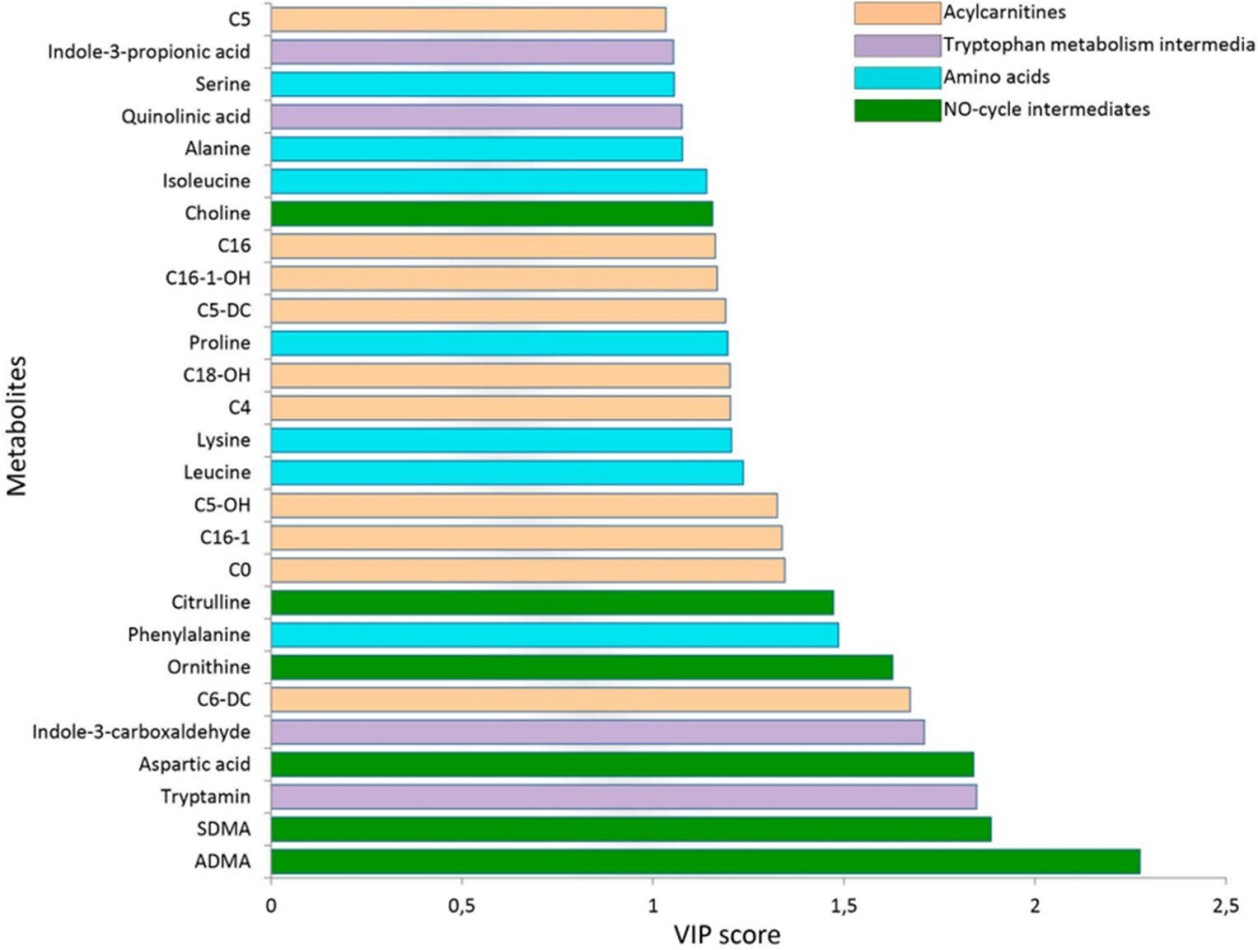
VIP score plot of the OPLS-DA model. The VIP score represents the overall contribution of a variable to the model and is calculated as a weighted sum of the squared correlations of the OPLS-DA components and the original variable.

For better specification of the metabolic alterations associated with NSCLC, the OPLS-DA model was built comprising one predictive and five orthogonal components (R2Xcum = 0.78; R2Y = 0.72; Q2 = 0.629) (Supplementary material, Fig. S3). Based on this model, VIP scores were calculated and later used for the analysis of the loadings reflecting the effect of each variable on the response^18^. Thus, 27 metabolites with VIP > 1 were selected as significant (Fig. 2).

**Figure 3:**
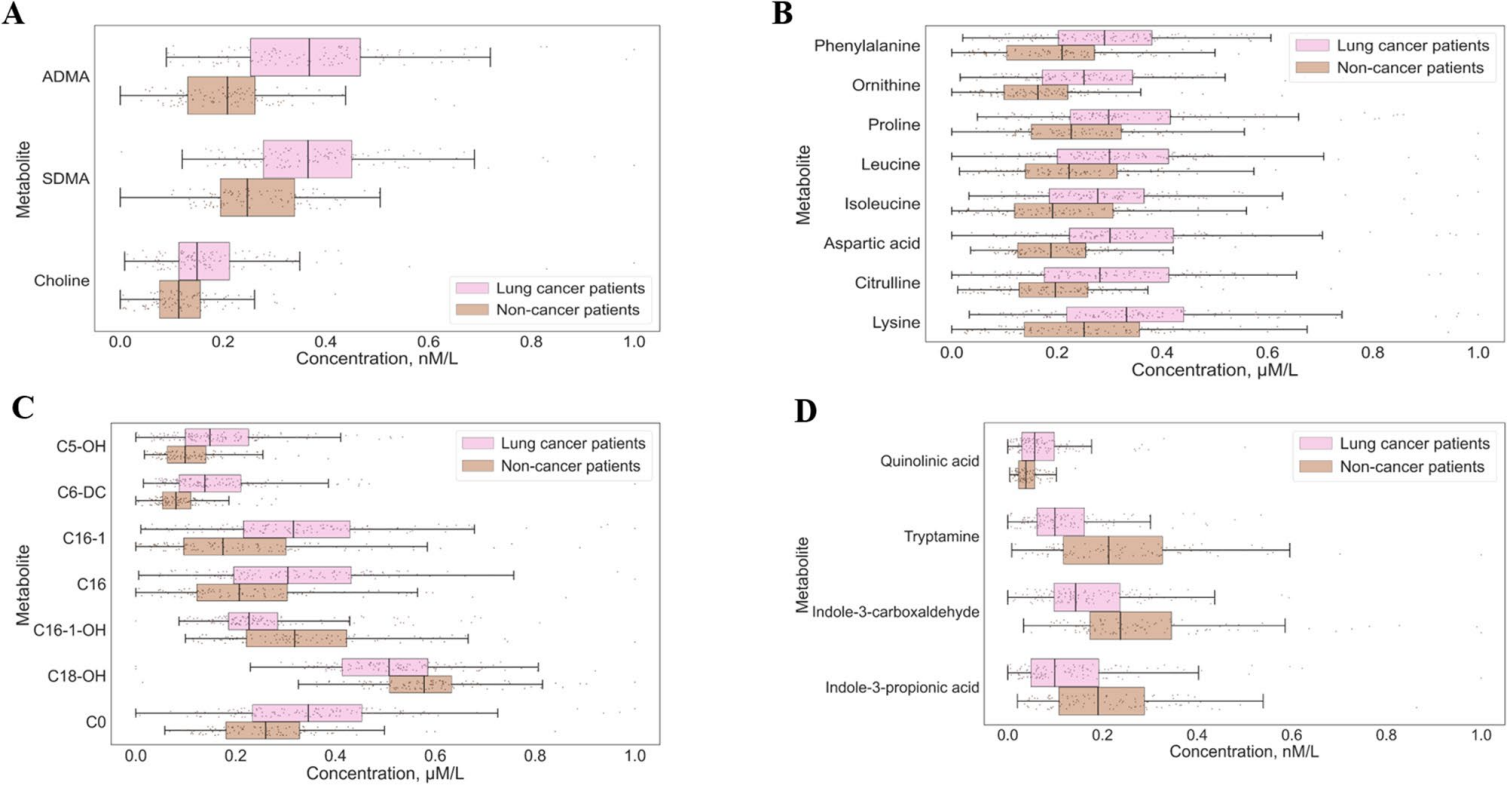
Box-plots of the preliminary selected significantly changed metabolites: (**A**)—NO-cycle intermediates; (**B**)—amino acids; (**C**)—Acylcarnitines; (**D**)—tryptophan metabolism intermediates. *p-value < 0.05, **p-value < 0.01, ***p-value < 0.001.

Table 2 represents the information on the 27 significantly altered metabolites that differed between NSCLC and NC patient groups and was extracted based on their FDR-corrected p value, AUCROC, Youden index and VIP score. The box plots of these **significantly changed metabolites are depicted in Fig. 3a–d.

**Table 2:** Significantly altered metabolites with corresponding AUC scores, Younden index, P-value and VIP-score.

	Metabolite	Metabolomic panel	AUC score	Younden index	P-value	VIP score
1	ADMA	Urea cycle	0.80	0.51641	< 0.00001	2.27
2	Asp	Amino acids	0.76	0.58881	< 0.00001	1.84
3	C6-DC	Acylcarnitines	0.76	0.54105	< 0.00001	1.67
4	Tryptamine	Tryptophan metabolism	0.76	0.54709	< 0.00001	1.85
5	SDMA	Urea cycle	0.75	0.46616	< 0.00001	1.88
6	Indole-3-carboxaldehyde	Tryptophan metabolism	0.74	0.64726	< 0.00001	1.71
7	Orn	Amino acids	0.73	0.61618	< 0.00001	1.63
8	Phe	Amino acids	0.70	0.5775	< 0.00001	1.48
10	C16-1	Acylcarnitines	0.69	0.54058	< 0.00001	1.34
11	Cit	Amino acids	0.69	0.58911	< 0.00001	1.47
12	Indole-3-propionic acid	Tryptophan metabolism	0.69	0.615	< 0.00001	1.05
13	C5-OH	Acylcarnitines	0.68	0.54112	< 0.00001	1.32
15	C0	Acylcarnitines	0.68	0.59459	< 0.00001	1.34
16	Leu	Amino acids	0.68	0.50.641	< 0.0001	1.24
17	C16-1-OH	Acylcarnitines	0.67	0.54207	< 0.0001	1.17
18	Ile	Amino acids	0.67	0.53259	< 0.0001	1.14
19	C16	Acylcarnitines	0.66	0.53065	< 0.0001	1.16
20	C18-OH	Acylcarnitines	0.66	0.54106	< 0.0001	1.20
21	Quinolinic acid	Tryptophan metabolism	0.66	0.56592	< 0.0001	1.07
22	Choline	Urea cycle	0.66	0.50.109	< 0.001	1.16
23	Lysine	Amino acids	0.65	0.52766	< 0.001	1.20
24	Proline	Amino acids	0.65	0.49426	< 0.001	1.19

### Correlation network analysis

Additionally, correlation network analysis of the received results was conducted to facilitate nonlinear interconnections between the metabolites. Debiased sparse partial correlation (DSPC) was applied to lung cancer and noncancer patients separately and performed by Cytoscape^19^. The data matrix with experimental measurements was uploaded into the Correlation Calculator. The program performs auto scaling and Pearson’s correlation analysis. Data were selected by setting a Pearson’s correlation coefficient threshold ± 1 and passed to DSPC. The result was visualized in Metscape^20^. The width of edges is based on adjusted p- values with a range of 0 — 0.2.

**Figure 4:**
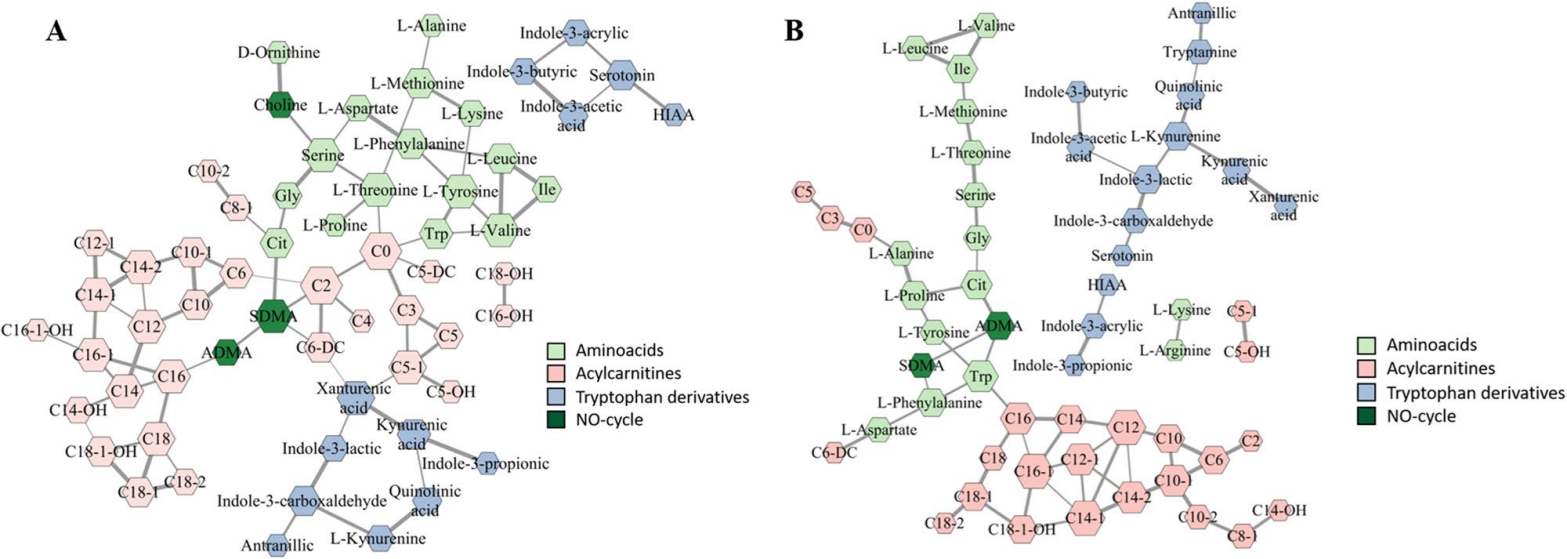
(**A**)—Correlation network obtained by DPSC algorithm using metabolites from lung cancer patients. (**B**)—Debiased Sparse Partial Correlation network constructed by using metabolites of the non-cancer patients. The size of the node represents the number of connections with other nodes. Width of edges is based on adjusted p-values. Colors of nodes are related to the corresponding metabolic panels of the metabolites.

Figure 4A,B represent the results of the Debiased Sparse Partial Correlation Network analysis of the profiled metabolites in the NC and NSCLC patients, respectively. Thus, the nodes correspond to the metabolites, and edges connect nodes if the corresponding metabolites are related.

In accordance with the correlation network analysis, we identified four modules of metabolites in the diseased group of patients and five modules in the noncancer patient group (the modules are matched with a stroke line). Moreover, it was found that the main hub metabolites in the diseased group included C0, C2, serine, threonine, xanthurenic acid, SDMA and serotonin. Additionally, based on the conducted correlation analysis, significantly altered ratios of the metabolites were found (Table 3).

**Table 3:** Significantly altered ratios of the metabolites identified through the correlation analysis.

Metabolite	AUC score	Younden index	p-value
serotonin/HIAA	0.77	0.623387	< 0.00001
Xanthurenic acid/C6-DC	0.74	0.620732	< 0.00001
Serine/Aspartic acid	0.72	0.598536	< 0.00001
Tyrosine/tryptophan	0.71	0.534586	< 0.00001
Phenylalanine/Tyrosine	0.70	0.539697	< 0.00001
Threonine/C0	0.70	0.598083	< 0.00001
Xanthurenic acid/C5-1	0.69	0.597235	< 0.00001
Threonine/Proline	0.69	0.525804	< 0.00001
SDMA/C2	0.67	0.539809	< 0.00001
Citrulline/C8-1	0.66	0.514331	< 0.0001

**Figure 5:**
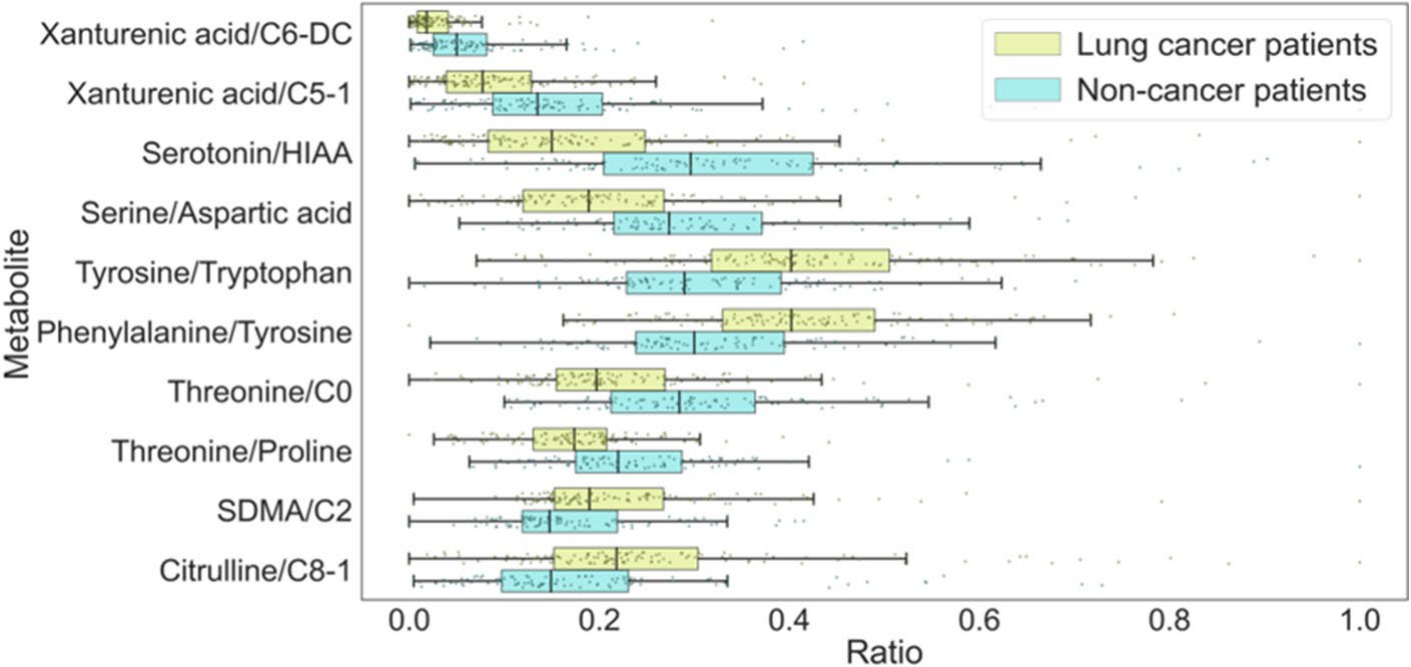
Ratios of the metabolites that significantly distinguished the NSCLC and the NC patient groups.

The boxplots of the significantly altered ratios are presented in Fig. 5.

The diagnostic performance of single metabolites does not usually match high levels of accuracy due to the strong biological variability of their absolute concentrations in blood. Therefore, to develop sensitive and accurate clinical prediction models, more comprehensive approaches need to be utilized. For this purpose, machine learning (ML) tools represent a unique opportunity for the development of supervised classification models based on the overall quantitative assessment of the key metabolic alterations in the studied biological fluids.

ML algorithms such as random forest, logistic regression, gradient boosting or support vector machine represent a viable alternative to a multivariate OPLS-DA method in the analysis of metabolomics data. In the present study, we assessed the diagnostic performance of the supervised ML-based models based on the following:

- the absolute concentrations of the preliminarily selected metabolites (Table 2);
- the absolute concentrations of the preliminarily selected metabolites (Table 2) and metabolite ratios (Table 3).

For each dataset, different ML-based algorithms were applied, including logistic regression, random forest (RF), support vector classifier (SVC), and gradient boosting classifier (GB).

The performance of the models was tested through the application of different quality control metrics: accuracy, confusion matrix, AUCROC, sensitivity and specificity. The results of the assessment for the applied ML algorithms using the datasets consisting of the selected metabolites and selected metabolites with the selected metabolic ratios are presented in Tables 4 and 5, respectively.

**Table 4:** Quality metrics of the ML-algorithms applied to the selected metabolites.

Algorithm	Accuracy	Confusion matrix (tp, fp, tn, fn)		AUC ROC	Sensitivity	Specificity
Logistic regression *('C'—1.44, 'penalty'—'l1', 'solver'—'liblinear')*	0.79	95	23	0.88	0.80	0.80
		23	77			
Support vector machine *(C'—1, ‘gamma'—0.1)*	0.78	102	32	0.86	0.86	0.76
		16	68			
Random forest *('criterion': 'entropy', 'max_depth': 10, 'max_features': 'log2', 'n_estimators': 100)*	0.77	94	24	0.87	0.80	0.80
		24	76			
Gradient boosting *(‘learning_rate' : 0.5, 'loss': 'exponential', 'max_features': 'log2', 'n_estimators': 50, 'subsample': 0.6)*	0.76	93	28	0.83	0.79	0.77
		25	72

**Table 5:** Quality metrics of the ML-algorithms applied to the selected metabolites with ratios.

Algorithm	Accuracy	Confusion matrix (tp fp fn tn)		AUC ROC	Sensitivity	Specificity
Logistic regression *{'C': 6.16, 'penalty': 'l1', 'logreg__solver': 'liblinear'}*	0.88	107	16	0.95	0.91	0.87
		11	84			
Support vector machine *{'C': 1000.0, 'gamma': 0.001, 'kernel': 'sigmoid'}*	0.84	102	20	0.92	0.86	0.84
		16	80			
Random forest *{‘criterion': 'gini', 'max_depth': 3, 'max_features': 'sqrt', 'n_estimators': 100}*	0.80	98	20	0.91	0.83	0.83
		20	80			
Gradient boosting *{'learning_rate': 0.1, 'loss': 'exponential', 'max_features': 'sqrt', 'n_estimators': 30, 'subsample' : 0.3}*	0.85	98	19	0.94	0.83	0.84
		20	81			

**Figure 6:**
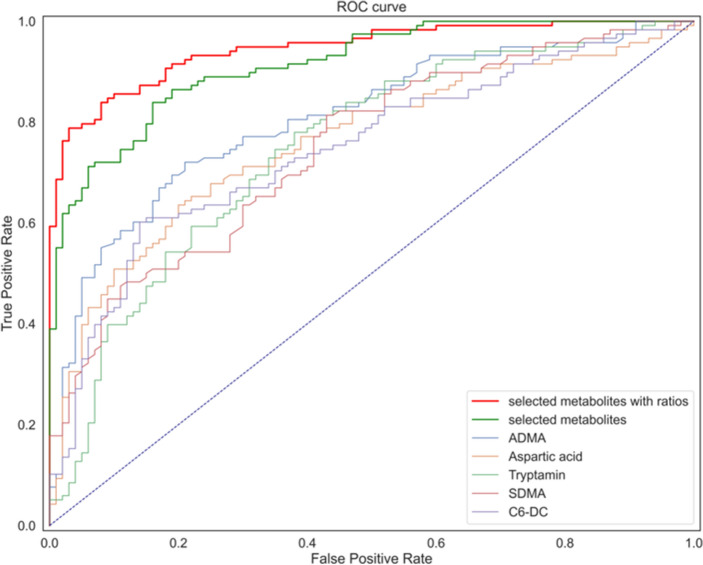
AUC ROC curves of the built models and individual metabolites. The ML model consisting of the selected significantly altered metabolites and significant ratios provides the best AUC value.

In both datasets, an algorithm of regularized logistic regression was found to be the most appropriate (Supplementary material, Fig. S4, S5). In general, binary classification models based on logistic regression algorithms commonly utilize the sigmoid function to a linear equation that outputs a range of values between 0 and 1 and are later used for dividing data into two classes. Furthermore, to determine the best diagnostic panel, we compared the diagnostic accuracy of the single top significant metabolites extracted through univariate analysis, as well as the two logistic regression models described above. Figure 6 illustrates a comparison of the AUC ROC curves of the built models and individual metabolites.

The AUC value of the model comprising the selected metabolites and their metabolite ratios was 0.95, whereas the model including only the selected metabolites was 0.88. At the same time, the AUC values of the individual most significantly altered metabolites were between 0.75 and 0.80.

## Discussion

Uncertainty in the etiology and mechanisms of lung cancer forces researchers from the biomedical field to develop novel strategies for deeper investigation. Metabolomics, as one of the OMIC technologies, provides an opportunity to uncover complex tumor-associated changes in a wide variety of quantitatively measured small molecules using a data-driven profiling strategy.

Because the sample size of the presented metabolomic study was relatively small, especially in comparison with the number of quantified metabolites, we assessed the metabolic pathway/set-wide differences between NSCLC patients and noncancer individuals to obtain our results. There were two complementary analyses consisting of a novel agnostic approach using correlation network analysis and common analysis that incorporates common knowledge on metabolic pathways.

Study of the biological role of the metabolites included in the final novel metabolic panel makes it possible to uncover new aspects and patterns of lung cancer and provides deeper characterization of the mechanisms associated with disease progression.

Meaningful differences in concentration levels of the amino acids found in serum samples of patients with NSCLC compared to the NC individuals included changes in the levels of aspartic acid, leucine, isoleucine, phenylalanine, citrulline and ornithine. Generally, such perturbations in the concentration of blood amino acids characterize tumor growth and proliferation^21^. The identified changes in the amino acid profile were mainly associated with the Krebs cycle and acetyl coenzyme A. The Krebs cycle is one of the central elements of cell biosynthesis.

Carnitine and acylcarnitines play a significant role in the energy and mitochondrial metabolism of fatty acids as well as in the regulation of free coenzyme A^22^. Carnitine and acylcarnitines are involved in the transport of fatty acids for β-oxidation in the mitochondrial matrix. Previously, abnormal amplification of fatty acid β-oxidation was found in NSCLC patients, presumably reflecting the proliferation, survival, drug resistance and metastasis processes of cancer cells^23^. At the same time, while short-chain fatty acids may freely diffuse through the interior side of the mitochondrial membrane, middle- and long-chain fatty acids are transported by acylcarnitines. Metabolic reprogramming of cancer cells affects acylcarnitine concentrations in blood. Cancer cells utilize acylcarnitines for the regulation of energy and synthesis of metabolic intermediates for more rapid proliferation^24^. Therefore, the overall tendency for the elevation of long-chain acylcarnitines in NSCLC patient blood compared to that of NC individuals identified in the present study may be associated with the enhanced β-oxidation of fatty acids. Despite the fact that acylcarnitine concentration in plasma does not fully reflect its metabolism in tissues, it apparently represents the block or inhibition of the overall fatty acid circulation in the body, which in this case is not controlled by the common homeostasis mechanisms. Interestingly, lower levels of hydroxylated long-chain acylcarnitines were identified in NSCLC patients than in noncancer individuals. This may characterize inhibition of hydroxylation in long-chain acylcarnitines that needs further exploration. At the same time, short-chain acylcarnitines significantly altered in the presented research are known as intermediates of BCAA metabolism and are also strongly affected in NSCLC patients.

The arginine biosynthetic pathway plays a crucial role in different pathophysiological mechanisms, such as cell signaling, immune response and protein synthesis^31–33^. In the present study, arginine pathway intermediates, including ornithine, citrulline, ADMA and SDMA, were significantly elevated in the blood of NSCLC patients. ADMA and SDMA are typically synthesized during protein breakdown and act as inhibitors of arginine synthesis. At the same time, increased concentrations of ADMA and SDMA were found in endothelial dysfunction associated with different cancers and chronic diseases^34^. Other significantly changed metabolites—citrulline and ornithine—take part in the urea cycle serving for the conversion of ammonia into urea, as well as acting as transcriptome reprogramming in cancer metabolism^25^. Thus, we hypothesize that significant accumulation of ornithine and citrulline in the plasma of NSCLC patients is associated with dysregulation in the urea cycle. As a result, during carcinogenesis, dysregulated metabolic pathways may promote tumor survival and growth.

Intermediates of tryptophan metabolism provide complex and multifaceted effects on lung cancer cells as well as on cancer-associated cells in immune escape. These metabolites are responsible for oxidative stress and inflammation. Thus, in the present study, the accumulation of quinolinic acid in the blood of NSCLC patients may be explained by its ability to inhibit T and natural killer cell proliferation, which promotes tumor growth^26^. At the same time, xanthurenic acid, another downstream metabolite of the kynurenine pathway, was downregulated in NSCLC patients, which complies with a previously conducted study on NSCLC patients^27^. Indole tryptophan derivatives (indole-3-carboxaldehyde and indole-3-propionic acid) related to gut microbiota were significantly lowered in the blood of the NSCLC group. Previously, numerous studies showed a direct strong association of gut microbiota with the risks and development of lung cancer^28,29^.

**Figure 7:**
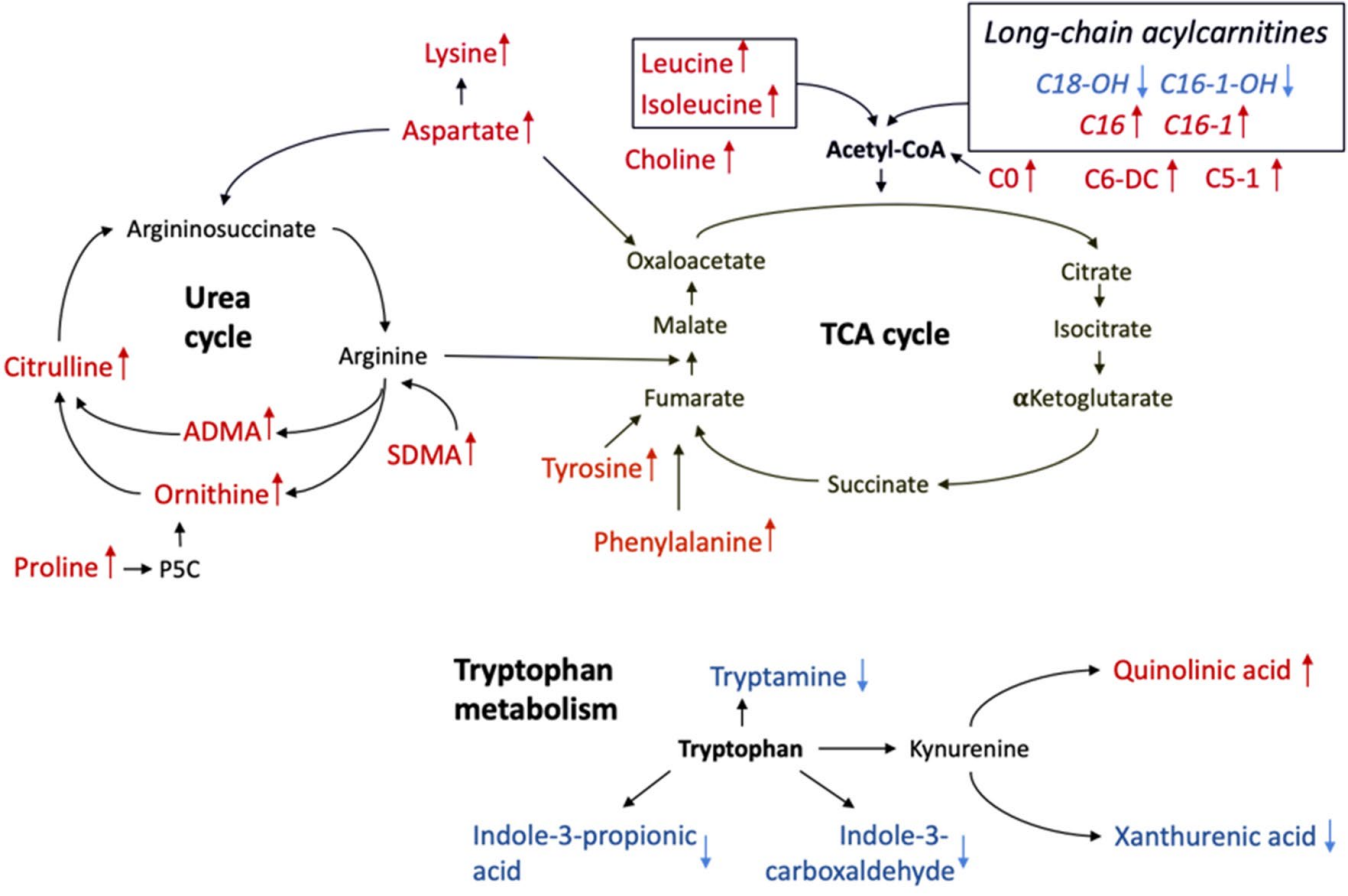
Scheme of the significantly altered metabolites and metabolic pathways in blood samples of the NSCLC patients compared to the NC individuals.

Overall elevation of the selected metabolites presumably represents novel therapeutic targets for the treatment of patients with NSCLC. Figure 7 illustrates a schematic overview of the interconnections of all significantly changed metabolites measured in blood samples of the NSCLC patients compared to that of the NC individuals.

In general, the metabolism of biological systems is usually represented as a network of metabolites, whose interconnections are predominantly based on enzymatic reactions that catalyze their interconversions^30^. However, through application of graph theory, such metabolic networks may be utilized not only for the visualization of the main biochemical pathways involved in the pathogenesis of the disease but also for interpretation of nonintuitive mechanisms and structures of metabolic relationships.

While application of the univariate and multivariate analyses serves to identify metabolites that better discriminate the studied groups, in many cases they analyze perturbations only in fragmented parts of the whole system that may result in the loss of the important nonlinear information extracted from the complex metabolomic data. The information gained from the correlation network analysis may show associations between distantly located (from a biochemical point of view) metabolites. It may underline complex and full information on interactions among metabolic pathways and associated biological effects on a systematic level. Moreover, this method allows the identification of highly connected hub metabolites (nodes) that may more likely become new relevant biological markers.

In the present study, correlation network analysis was performed using the DSPC method, which demonstrated significantly different interconnections of the analyzed metabolites. We may underline the relatively unchanged connections in medium- and long-chain acylcarnitines and branched chain amino acids (valine, leucine, isoleucine) between NSCLC and NC patients. At the same time, major perturbations were found in the amino acid profile, where in the diseased group, the number of new interconnection bonds was significantly altered, forming two main hubs—serine and threonine. We hypothesize that carnitine (C0) and acetylcarnitine (C2) play crucial roles in the development of NSCLC, as they become main metabolic hubs connecting tryptophan metabolism intermediates, long-chain acylcarnitines and amino acids in the lung cancer group. According to the correlation analysis, several changes were also found among tryptophan catabolites. Thus, whereas in the NC patients, metabolites from this panel were separated from other modules, in the case of NSCLC, they showed a strong correlation with short-chain acylcarnitines through the connection with xanthurenic acid. Moreover, the concentration ratios of xanthurenic acid with C5-1 and C6-DC were found to be significant and were further utilized for diagnostic model development. As a result, the application of correlation analysis reflects new significant ratios of metabolites, most of which did not have significant differences in their absolute values.

The classification models obtained through the application of machine learning methods utilize the discriminative abilities of multiple combinations of metabolites. Robust and consistent ML models may provide new biological interpretations of the disease through enhanced and deep statistical analysis of the NSCLC and NC patient metabolome. Thus, the presented analysis provides an assessment of the predictive power of biofluid metabolomics for machine learning-based diagnostics of NSCLC. We compared the diagnostic accuracy of the individual most significantly altered metabolites (ADMA, aspartic acid, tryptamine, SDMA, C6-DC) with the diagnostic accuracy of the metabolic panel comprising multiple combinations of the selected metabolites and the metabolic panel comprising multiple combinations of the selected metabolites and concentration ratios based on correlation network analysis. We may conclude that the best diagnostic accuracy was achieved for the metabolomic panel of the selected metabolites and metabolic ratios. The diagnostic accuracy of the logistic regression method was relatively higher than that of the other four supervised ML methods across the investigated quality metrics.

We have demonstrated that the ML model using absolute concentration values of the selected metabolites and metabolite ratios provided the best diagnostic accuracy in NSCLC patients. Single biomarkers may not have the ability to accurately diagnose NSCLC, whereas ML tools that use tentatively selected metabolites and their ratios significantly enhance the specificity of diagnostics.

Utilisation of the developed metabolic panel certainly needs to be validated in external patient cohorts as well as between different stages of LC. Furthermore, in the present study we did not evaluate the clinical factors that could influence the results obtained, such as differences in the M/F ratio of the patient groups considered. However, the present study provides a novel metabolic panel with significant diagnostic capabilities that may be suitable for future preliminary screening tests. It is suggested that future studies should also focus on the processes associated with a deeper investigation of β-oxidation in mitochondria, with particular attention to fatty acid metabolism. In particular, the possible changes in the concentration of long-chain fatty acids in the blood of lung cancer patients and their correlation with the above-described changes in long-chain acylcarnitines could be addressed. Besides this, interesting findings have recently been made in relation to mitochondrial β-oxidation and deuterium depletion. It was found that the ratio of deuterium/hydrogen isotopes (2 H/1 H) may influence abnormal cell proliferation and tumour progression^35^. For example, the results of the in vivo study showed that a low concentration of deuterium in water is able to inhibit lung tumour growth^36^. In addition, the replacement of normal water with deuterium-depleted water (DDW) contributed to an increase in median survival time in patients with prostate cancer^37^. Long-chain fatty alcohols as metabolites were observed to be associated with survival in patients with lung cancer, possibly due to the low deuterium content of ketogenic substrates^38^. The resulting oxidation of ketogenic substrates in the low deuterium water of the TCA cycle promotes the subsequent synthesis of DNA with more stable hydrogen bonds^39^.

## Conclusions

The developed machine learning lung cancer model may serve as a prototype for timely diagnostics of lung cancer that may be introduced in routine clinical use in the future. Overall, we have demonstrated that the combination of metabolomics and up-to-date bioinformatics can be used as a potential tool for proper diagnostics of patients with NSCLC.

## Supplementary Information

Supplementary Information 1.

Supplementary Information 2.

## Data Availability

All data generated or analyzed during this study are included in this published article, please consult Supplementary Information files.
