# Supplementary material for: Targeted metabolomic profiling as a tool for diagnostics of patients with non-small-cell lung cancer
Source: Sci Rep. 2023 Jul 8;13:11072. doi: 10.1038/s41598-023-38140-7 (PMC10329697; doi:10.1038/s41598-023-38140-7)
Supplement: Supplementary file 1 — Supplementary Information 1. [file 41598_2023_38140_MOESM1_ESM.docx]

**Supplementary information**

**Targeted metabolomic profiling as a tool for diagnostics of patients with non-small-cell lung cancer**

Ksenia M. Shestakova (1), Natalia E. Moskaleva (1), Andrey A. Boldin (2,3), Pavel M. Rezvanov (2,3), Alexandr V. Shestopalov (4), Sergey A. Rumyantsev (4), Elena Yu. Zlatnik (5), Inna A. Novikova (5), Alexander B. Sagakyants (5), Sofya V. Timofeeva (5), Yuriy Simonov (2), Sabina N. Baskhanova (1) , Elena Tobolkina (6)*, Serge Rudaz (6) and Svetlana A. Appolonova (2,3)

1. World-Class Research Center Digital Biodesign and Personalized Healthcare, I.M. Sechenov First Moscow State Medical University, 119435 Moscow, Russia
2. Laboratory of Pharmacokinetics and Metabolomic Analysis, Institute of Translational Medicine and Biotechnology, I.M. Sechenov First Moscow Medical University, 119435 Moscow, Russia
3. I.M. Sechenov First Moscow State Medical University, 119435 Moscow, Russia.
4. Pirogov Russian National Research Medical University, Moscow, 117997 Russia
5. National Medical Research Centre for Oncology (Rostov-on-Don, Russia). 344019, Rostov-on-Don, 14 Liniya, 63
6. Institute of Pharmaceutical Sciences of Western Switzerland, University of Geneva, 1206 Geneva, Switzerland

*Correspondence should be addressed to the following author(s):

Dr. Elena Tobolkina

Institute of Pharmaceutical Sciences of Western Switzerland,

University of Geneva,

Geneva 4, Switzerland

elena.tobolkina@unige.ch

Table S1. The list of the analyzed metabolites during the performed metabolomic screening

| Profiling | Metabolites |
| --- | --- |
| Amino Acid profiling | Glycine (Gly), alanine (Ala), proline (Pro), valine (Val), leucine (Leu), isoleucine (Ile), ornithine (Orn), aspartate (Asp), phenylalanine (Phe), arginine (Arg), citrulline (Cit), serine (Ser), threonine (Thr), lysine (Lys), tryptophan (Trp), tyrosine (Tyr), methionine (Meth) |
| Asymmetric Dimethylarginine, Symmetric Dimethylarginine and Сholine | ADMA, SDMA, Choline |
| Acylcarnitine profiling | Carnitine (C0), acetylcarnitine (C2), propionylcarnitine (C3), butirylcarnitine (C4), tiglycarnitine (C5-1), isovalerylcarnitine (C5), hexanoylcarnitine (C6), hydroxyisovalerylcarnitine (C5-OH), glutarylcarnitine (C5-DC), octenoylcarnitine (C8-1), adypoylcarnitine (C6-DC), decadienoylcarnitine (C10-2), decenoylcarnitine (C10-1), decanoylcarnitine (C10), dodecenoylcarnitine (C12-1), dodecanoylcarnitine (C12), tetradecadienoylcarnitine (C14-2), tetradecenoylcarnitine (C14-1), tetradecanoylcarnitine (C14), hydroxytetradecanoylcarnitine (C14-OH), palmitoleylcarnitine (C16-1), palmitoylcarnitine (C16), hydroxyhexadecenoylcarnitine (C16-1-OH), hydroxyhexadecanoylcarnitine (C16-OH), linoleylcarnitine (C18-2), oleyoylcarnitine (C18-1), stearoylcarnitine (C18), hydroxyoleyoylcarnitine(C18-1-OH), hydroxystearoylcarnitine (C18-OH) |
| Tryptophan metabolites prifling | Kynurenine, Serotonin, Quinolinic acid, HIAA, Tryptamin, Antranillic acid, Indole-3-lactic acid, Indole-3-acetic acid, Indole-3-carboxaldehyde, Indole-3-acrylic acid, Indole-3-propionic acid, Indole-3-butyric acid, Xanturenic acid, Kynurenic acid |

A.


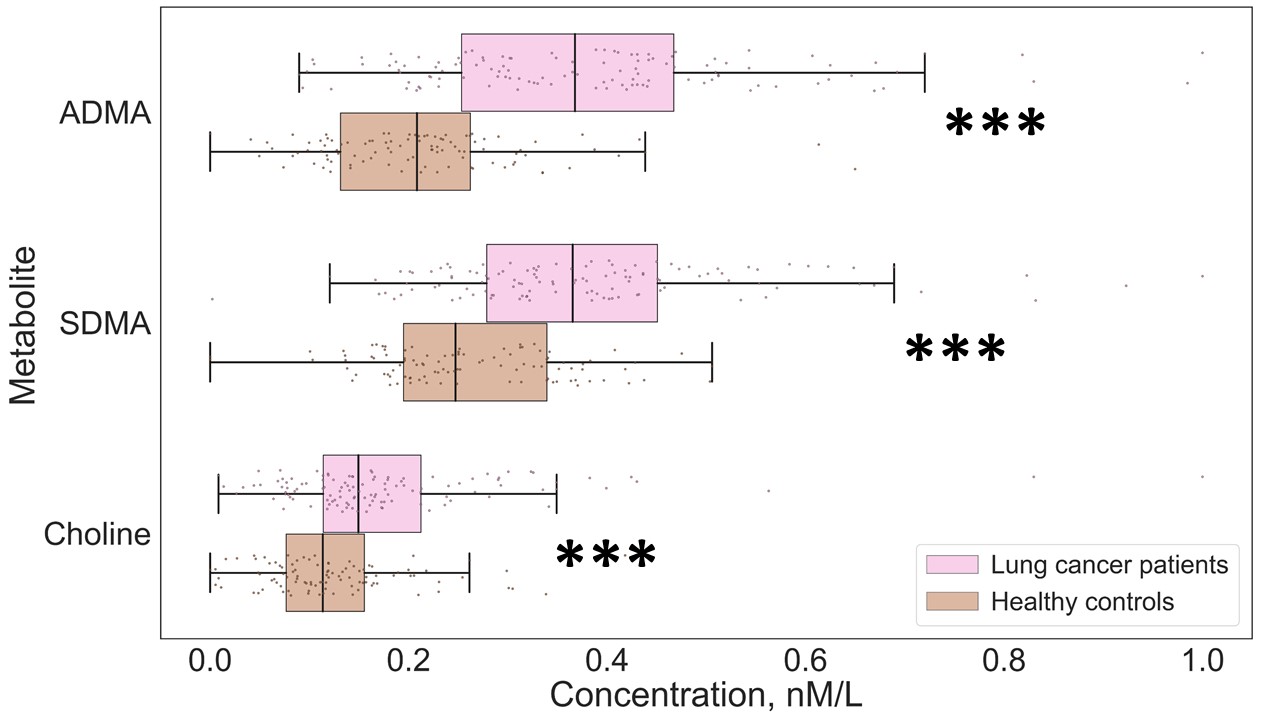


B.


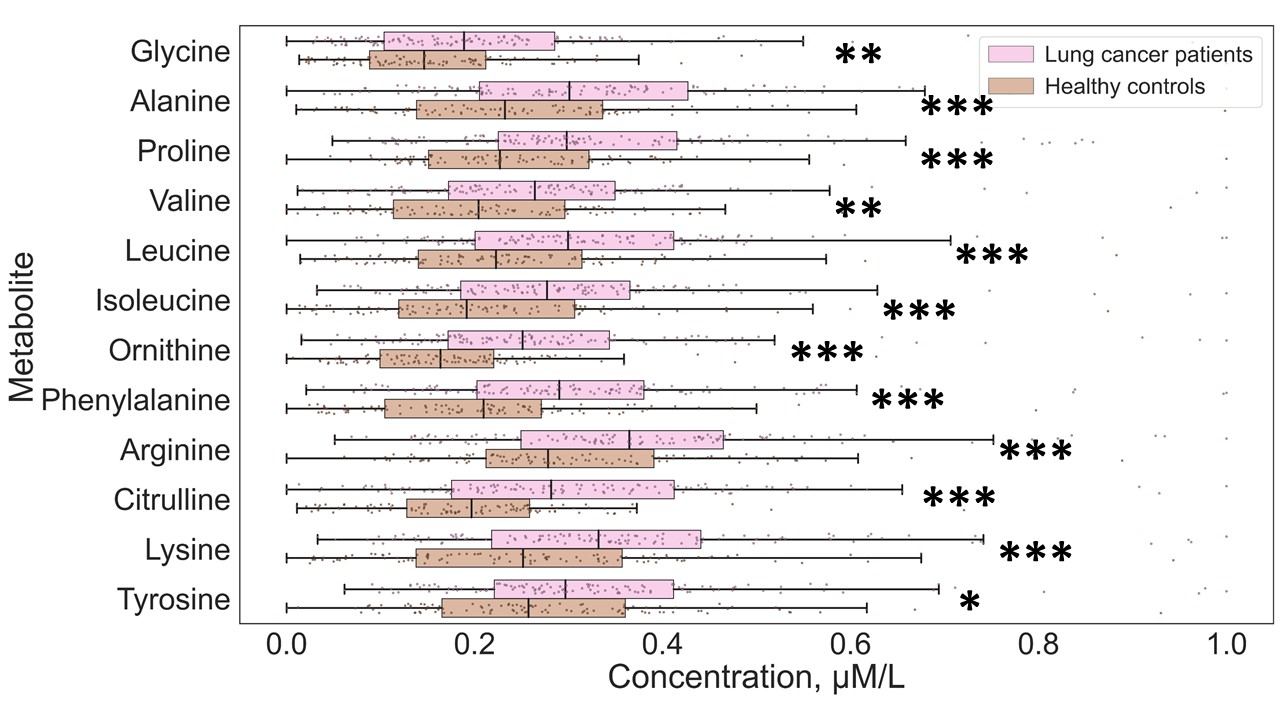


C.


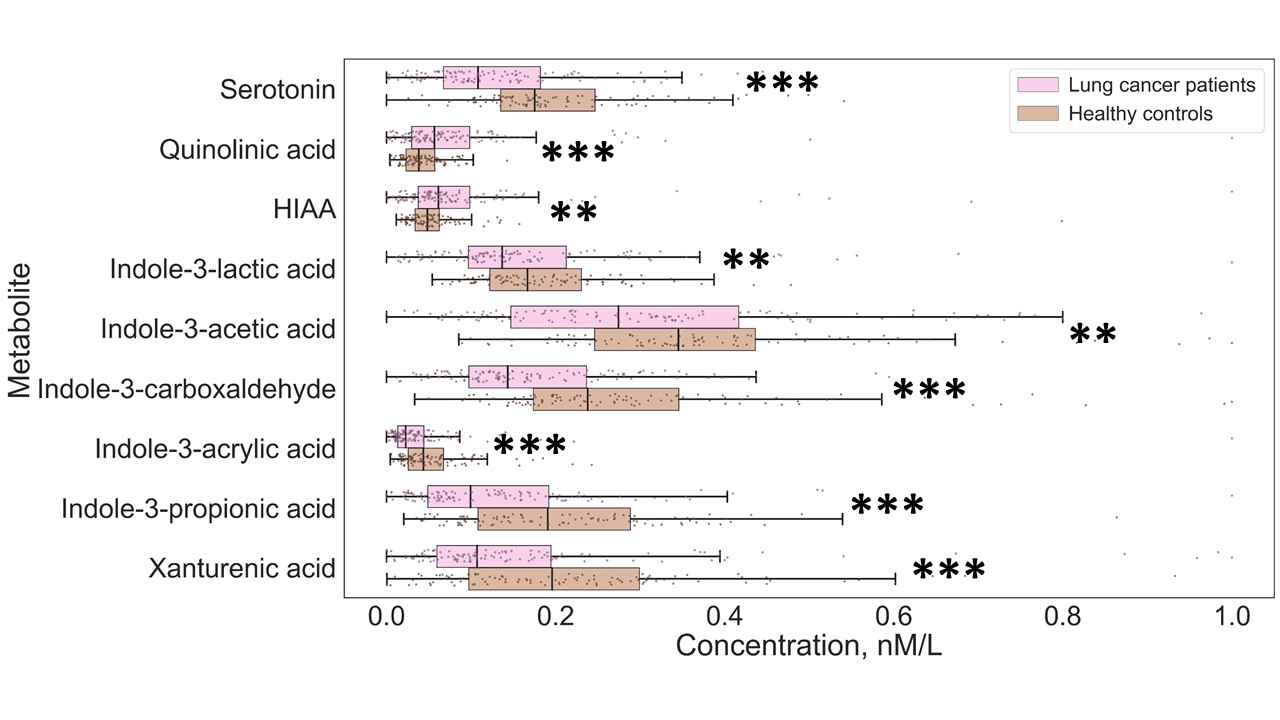


D.


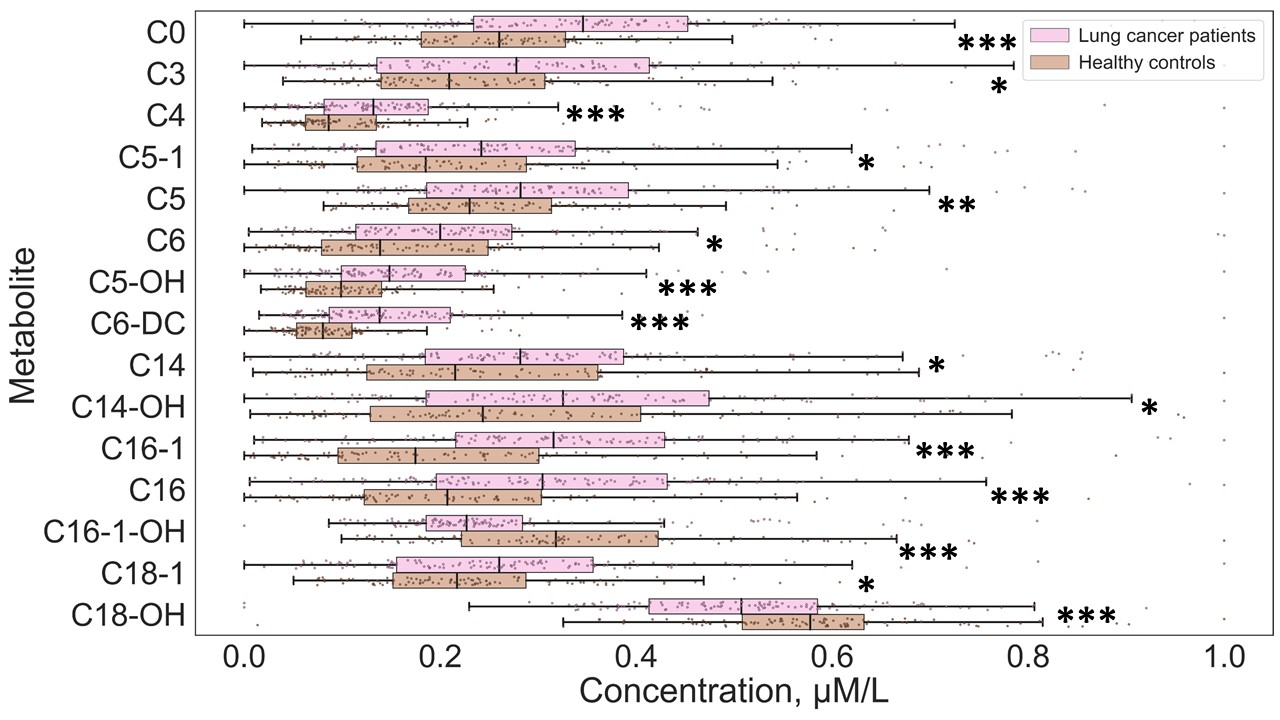


Figure S1. Box-plots of the significantly altered metabolites in serum samples of the patients with NSCLC and the NC individuals, related to: А - NO-cycle intermediates; B - amino acids; C - tryptophan metabolism intermediates; D - Acylcarnitines; * - p-value < 0.05, ** - p-value <0.01, *** - p-value < 0.001.


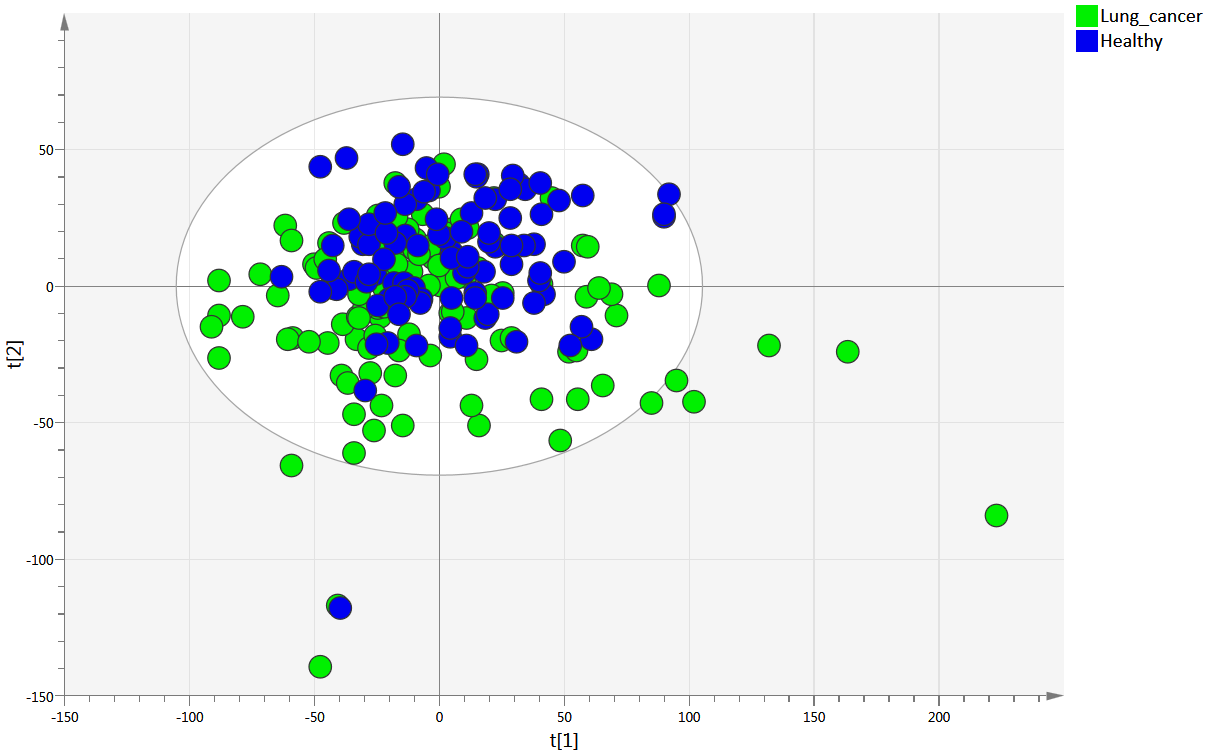


Figure S2. PCA score plot based on the results of the wide-scaled targeted metabolomic profiling


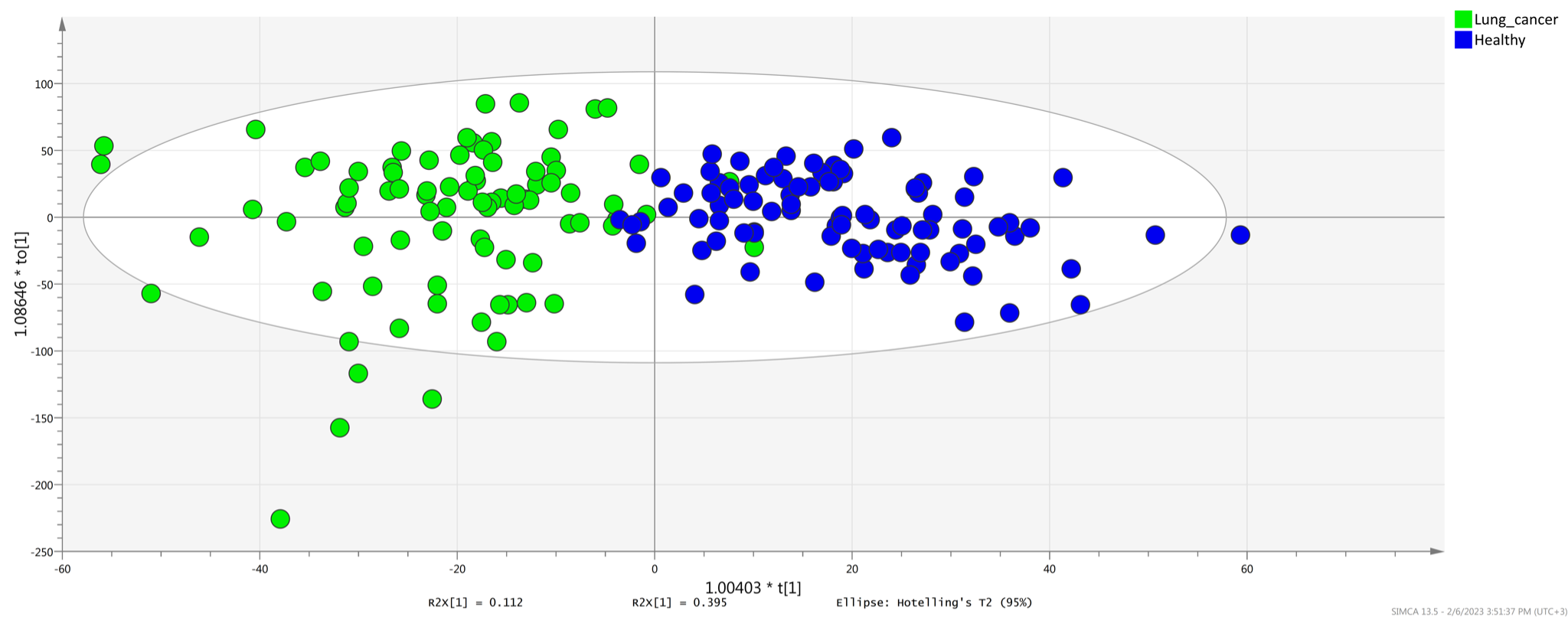


Figure S3. OPLS-DA scores plot for the model discriminating blood samples from NCLC patients and NC patients

**
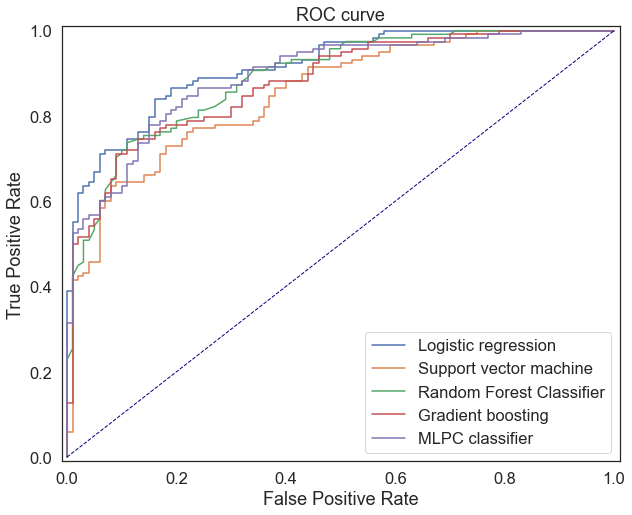
**

Figure S4. Comparison of the applied ML algorithms for the dataset of the absolute values of the selected metabolites and their ratios


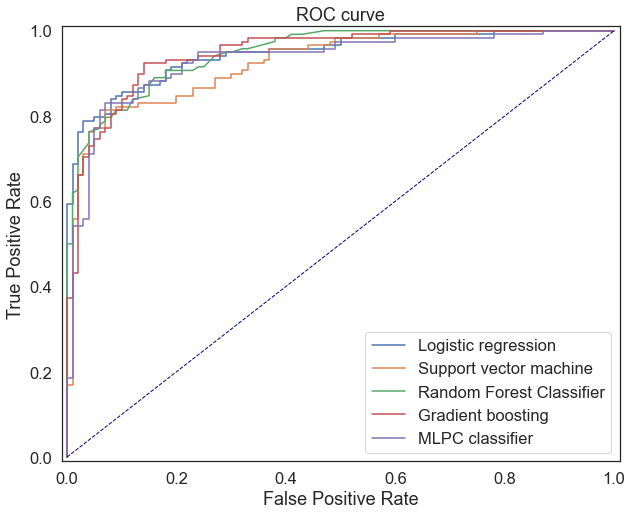


Figure S5. Comparison of the applied ML algorithms for the dataset of the absolute values of the selected metabolites and their ratios
